# Out-of-Field Doses Produced by a Proton Scanning Beam Inside Pediatric Anthropomorphic Phantoms and Their Comparison With Different Photon Modalities

**DOI:** 10.3389/fonc.2022.904563

**Published:** 2022-07-22

**Authors:** Željka Knežević, Liliana Stolarczyk, Iva Ambrožová, Miguel Á. Caballero-Pacheco, Marie Davídková, Marijke De Saint-Hubert, Carles Domingo, Kinga Jeleń, Renata Kopeć, Dawid Krzempek, Marija Majer, Saveta Miljanić, Natalia Mojżeszek, Maite Romero-Expósito, Immaculada Martínez-Rovira, Roger M. Harrison, Paweł Olko

**Affiliations:** ^1^ Ruđer Bošković Institute, Zagreb, Croatia; ^2^ Danish Centre for Particle Therapy, Aarhus, Denmark; ^3^ Institute of Nuclear Physics, PAN, Krakow, Poland; ^4^ Nuclear Physics Institute of the Czech Academy of Sciences, CAS, Řež, Czechia; ^5^ Universitat Autònoma de Barcelona, Bellaterra, Spain; ^6^ Belgium Nuclear Research Centre, Mol, Belgium; ^7^ Tadeusz Kosciuszko Cracow University of Technology, Cracow, Poland; ^8^ Skandion Clinic, Uppsala, Sweden; ^9^ University of Newcastle upon Tyne, Newcastle upon Tyne, United Kingdom

**Keywords:** scanning proton therapy, out-of-field doses, anthropomorphic phantoms, track detectors, RPL detectors, TL detectors, brain tumor irradiations

## Abstract

Since 2010, EURADOS Working Group 9 (Radiation Dosimetry in Radiotherapy) has been involved in the investigation of secondary and scattered radiation doses in X-ray and proton therapy, especially in the case of pediatric patients. The main goal of this paper is to analyze and compare out-of-field neutron and non-neutron organ doses inside 5- and 10-year-old pediatric anthropomorphic phantoms for the treatment of a 5-cm-diameter brain tumor. Proton irradiations were carried out at the Cyclotron Centre Bronowice in IFJ PAN Krakow Poland using a pencil beam scanning technique (PBS) at a gantry with a dedicated scanning nozzle (IBA Proton Therapy System, Proteus 235). Thermoluminescent and radiophotoluminescent dosimeters were used for non-neutron dose measurements while secondary neutrons were measured with track-etched detectors. Out-of-field doses measured using intensity-modulated proton therapy (IMPT) were compared with previous measurements performed within a WG9 for three different photon radiotherapy techniques: 1) intensity-modulated radiation therapy (IMRT), 2) three-dimensional conformal radiation therapy (3D CDRT) performed on a Varian Clinac 2300 linear accelerator (LINAC) in the Centre of Oncology, Krakow, Poland, and 3) Gamma Knife surgery performed on the Leksell Gamma Knife (GK) at the University Hospital Centre Zagreb, Croatia. Phantoms and detectors used in experiments as well as the target location were the same for both photon and proton modalities. The total organ dose equivalent expressed as the sum of neutron and non-neutron components in IMPT was found to be significantly lower (two to three orders of magnitude) in comparison with the different photon radiotherapy techniques for the same delivered tumor dose. For IMPT, neutron doses are lower than non-neutron doses close to the target but become larger than non-neutron doses further away from the target. Results of WG9 studies have provided out-of-field dose levels required for an extensive set of radiotherapy techniques, including proton therapy, and involving a complete description of organ doses of pediatric patients. Such studies are needed for validating mathematical models and Monte Carlo simulation tools for out-of-field dosimetry which is essential for dedicated epidemiological studies which evaluate the risk of second cancers and other late effects for pediatric patients treated with radiotherapy.

## 1 Introduction

Proton beam therapy offers a reduced entrance dose and a negligible exit dose when compared with photon irradiation techniques. The presence of the Bragg peak in proton therapy allows for better conformation of dose to the target and results in sparing of surrounding normal tissues and consequently can reduce the acute and late side effects of the treatment. Reducing the probability of short- and long-term complications of radiotherapy is of special importance when tumors are located next to the critical organs and while treating pediatric patients. In the past decades owing to new diagnostic procedures and continuous improvement and introduction of new treatment modalities, the probability of cancer cure and survival rate has risen considerably. In general, around 80% of children with malignant diseases are successfully treated with survival rates greater than 5 years (1). Central nervous system tumors such as gliomas, medulloblastoma, and ependymal tumors are the most common solid malignancies in childhood (30% of all pediatric tumors). Radiation therapy is an integral component of therapy for pediatric brain tumors. In recent years, the number of children, especially with brain tumors, treated using proton therapy has increased significantly (2–6). Improvement in the treatment outcome and the increase in the number of long-term survivors of child malignancies emphasize the importance of late radiation-induced effects. Due to a long-life expectancy after treatment, approximately 70% of children will develop some kind of short- or long-term treatment-related complications (7, 8). A multitude of radiation epidemiology studies have revealed the high prevalence of radiation-induced late effects including radiogenic secondary cancers (9, 10). The risk of developing secondary cancer following radiotherapy (years or decades after the treatment) is by a factor of 10 higher in children in comparison to adults and can be as high as 12% (7, 11–15). It depends upon multiple factors including patient age, size, biological and genetic predisposition of the individual, type of therapy received (chemotherapy and/or radiotherapy), the organ and tissue sites receiving radiation, and also the dose delivered during the treatment. Most existing risk models are designed for low-dose and low-dose-rate exposures and cannot be easily translated to radiotherapy, where dose is fractionated and organ doses may be heterogeneous (16). Therefore, dedicated epidemiology studies are required for pediatric exposures during radiotherapy. Such studies need accurate dosimetry input from experiments in combination with validated analytical models or Monte Carlo simulations. In the recent years, the continued technological expansion of radiotherapy has resulted in the use of advanced treatment modalities, such as proton radiotherapy, intensity-modulated radiation therapy (IMRT), volumetric-modulated arc therapy (VMAT), image-guided radiotherapy (IGRT), and magnetic resonance linear accelerators (MR-LINAC). These new techniques provide better dose distributions and are more conformal in comparison to the conventional ones. Nevertheless, they still produce scattered or secondary radiation in the interactions of a primary beam with treatment unit and patient body. Doses outside the treatment fields are much lower in comparison to the doses within the primary field, but they are of radiobiological interest as they are received by healthy organs and may lead to secondary cancer (17, 18). Treatment planning systems (TPS) commonly used to estimate dose distributions inside a patient body calculate doses to the target and organs in the proximity of the target with high accuracy. Outside the treatment field, in the region of out-of-field doses, TPS calculations become inaccurate and may even underestimate the dose by up to 40% (18–21). Moreover, dose calculations in remote organs are often restricted by the limited anatomical coverage of the computed tomography (CT) used for treatment planning. In proton therapy, the situation is even more complicated due to a complex spectrum of secondary neutrons as well as secondary gammas and scattered charged particles contributing to out-of-field doses (22). Neutrons are of particular concern due to their high relative biological effectiveness (RBE) and cannot be neglected in the evaluation of the potential risks (23, 24). The limitations in tracking of secondary radiation in most clinical treatment planning systems make measurements essential for out-of-field dose estimation. As doses in the out-of-field region vary with delivery technique, treatment site, field characteristics, and energy spectrum, measurements in this region are challenging. In proton radiotherapy, out-of-field doses are mostly evaluated based on measurements with track-etched detectors, bubble detectors, ionization chambers, and thermoluminescent detectors supported by in-room measurements with active detectors or Bonner Spheres and Monte Carlo simulations. It is worth pointing out that both energy and spatial distributions of secondary radiation can differ among different proton facilities. Therefore, it is important to model the specific beam and room geometries for Monte Carlo simulations of out-of-field doses. Such models should be validated against measurements. Most of the experiments described in the literature are aimed at measuring dose as a function of distance to the field (17, 18, 22, 25–27), and although such data are helpful for relative comparisons, information about organ doses is still missing in the literature. Moreover, in the published studies, experimental data for active scanning techniques are scarce as the majority of papers describe passive scattering techniques (14, 23, 26, 27). In the paper by Athar et al., out-of-field doses are simulated for an 8-year phantom and for different 6-MV IMRT plans and compared with passive and active proton therapy techniques (17). The results showed that at larger distances (25 cm and more) from the field edge, out-of-field organ doses are higher in IMRT than those in passive scattered proton therapy. For scanning proton beams, organ doses were lower (up to two orders of magnitude) in comparison to IMRT and also a proton passive scattering technique. In the paper by Ardenfors et al., organ doses from secondary radiation were calculated using MC simulations for an adult female patient and a 6-year pediatric patient for a proton spot scanning technique with different beam setups (25). The results showed that neutron equivalent doses for brain tumors treated with proton PBS are relatively low, of the order of mSv. In the publication by Gudowska et al., a literature review of the secondary doses to healthy tissues is given for different modern radiation therapy techniques (28). The review summarizes different methods of assessing secondary doses (MC simulations, TPS, measurements with different types of detectors). Doses were evaluated for organs in real patients or in different anthropomorphic and water phantoms. The data showed a large variation of secondary absorbed doses to healthy organs, ranging from ~0.007 mGy to 2.4 Gy per prescribed dose depending on the type and energy of the primary beam, irradiation technique, patient geometry, distance from the primary field tumor, and organ size.

In the literature, terminology on expressing secondary doses differs and it is not always clear how they are calculated and normalized and what radiation components are taken into account. In some studies, out-of-field doses are presented as absorbed doses, organ doses, or equivalent doses and one should be careful when comparing results within different studies. In addition, there is a variation with the target size and location but also type of phantom used, type of detector used, and their respective response in the secondary radiation field. Moreover, many studies focus only on one component of the secondary radiation field, namely, secondary neutrons, ignoring doses coming from secondary particles and secondary gamma radiation.

Since 2010, EURADOS Working Group 9 (Radiation Dosimetry in Radiotherapy) has been involved in the investigation of out-of-field radiation doses in photon and proton therapy especially in case of pediatric patients. Firstly, WG9 performed detailed characterization of the out-of-field doses, associated with proton PBS in a water phantom with both measurements and MC simulation, which clearly showed complexity associated with the secondary radiation field produced in proton PBS (22, 29–31). The next step included measurement campaigns organized by WG9 in the Centre of Oncology, Krakow University Hospital Centre Zagreb, and University Hospital Osijek to study secondary radiation for different photon radiotherapy techniques (32–34). Those experiments were followed by a measurement campaign which is presented in this paper dedicated to proton radiotherapy with pencil beam scanning technique (PBS) carried out at the Cyclotron Centre Bronowice IFJ PAN (Krakow, Poland) (29). In all experiments performed by WG9 for pediatric patients, in both photon and proton therapy, out-of-field organ doses were measured inside 5- and 10-year-old anthropomorphic phantoms for the same target size and location. This experimental consistency allows a direct comparison of out-of-field organ doses for different modalities of photon radiotherapy and proton PBS radiotherapy. Our studies were performed for a realistic clinical treatment of a pediatric brain lesion, to give a fair comparison between different treatment methods in a clinical scenario.

The main goal of this paper is to present out-of-field organ dose measurement results for 5- and 10-year-old anthropomorphic phantoms for the brain target irradiated with proton pencil beam scanning (PBS) technique. Both neutron and non-neutrons components of the secondary radiation field were taken into account. Results are compared with previously published data for the same clinical condition but for different photon radiotherapy techniques. Such comparison allows the potency of intensity-modulated proton therapy (IMPT) to reduce late radiation-induced effects to be evaluated.

## 2. Materials and methods

### 2.1. Intensity-Modulated Proton Therapy

#### 2.1.1 Experimental Setup

The irradiations were carried out at the Bronowice Cyclotron Centre (Krakow, Poland) with a pencil beam technique (PBS) at a dedicated scanning gantry (IBA Proton Therapy System - Proteus 235). Measurements of secondary gamma and neutron radiation were performed inside two anthropomorphic phantoms which represents 5- and 10-year-old children (CIRS phantom type 705D and type 706D ATOM, Computerized Imaging Reference Systems (CIRS), Inc., Norfolk, VA). Phantoms are made of tissue equivalent material and consist of 26 and 32 slices (each slice is 25 mm thick) with 180 and 213 detector holes for 5- and 10-year-old phantoms, respectively. Each slab contains holes of diameter 5 mm located within different organs. In this work, distance from the center of the dosimeter to the selected point within the phantom was used to characterize the out-of-field dose distribution for a given irradiation. These distances were calculated from CT images of the phantoms.

#### 2.1.2. IMPT Irradiation Plan

**Figure 1:**
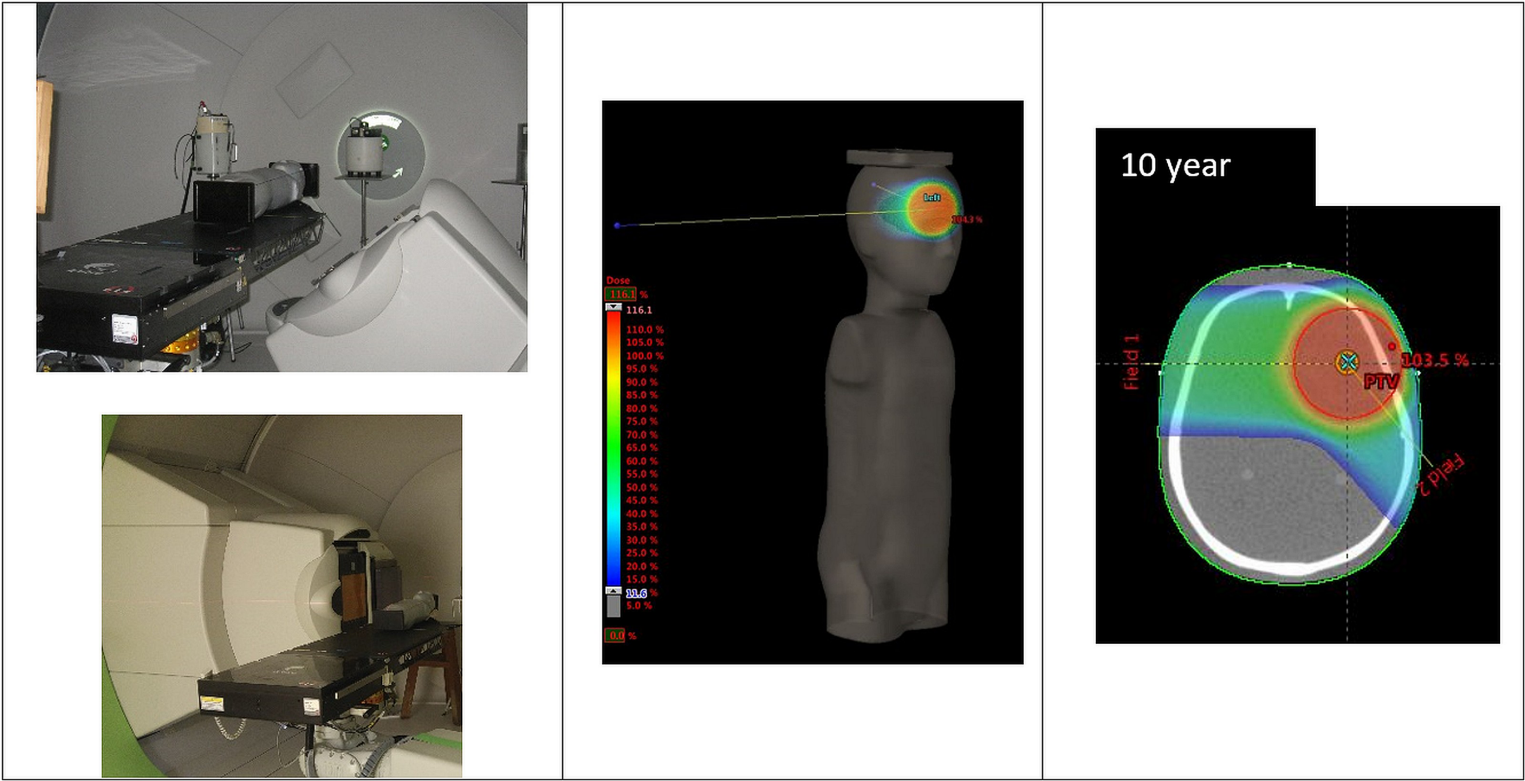
Irradiation setup and tumor location for the IMPT-simulated treatment.

Prior to irradiation, a CT (Siemens Somatom Definition AS Open) of each phantom was performed (2-mm slices, head first supine, FOV 500 mm). Radiotherapy plans were created using an IMPT treatment planning technique with an Eclipse v13.6 Treatment Planning System (TPS) (Varian). For both pediatric phantoms, treatment of a brain tumor was simulated. The planning target volume (PTV) comprises a 6-cm-diameter sphere (113 cm^3^) with the center on the left anterior side of the head (located in slice 3 as shown in **Figure 1**). In each case, the target was irradiated using two coplanar fields with gantry positioned at 140° and at 270° (**Table 1**). Phantoms were aligned at the treatment table in the supine position. The energy layers ranged from 70 to 140 MeV. No range shifter was used.

**Table 1:** IMPT plans parameters for 5- and 10-year-old phantoms.

Phantom	Field	Min. energy (MeV)	Max. energy (MeV)
5-year	F1 (270˚)	71.6	127.5
	F2 (140˚)	84.0	137.8
10-year	F1 (270˚)	70.5	128.2
	F2 (140˚)	99.2	144.6

For both phantoms, the planned physical dose to the target was *D*_T_ = 100 Gy for irradiation of luminescent dosimeters, *D*_T_ = 40 Gy for track-etched detectors. The applied dose was higher than usually used for actual treatments and was adapted to the sensitivity of the detectors in order to produce a signal above the detection threshold for detectors distant from the isocenter. The dose values for the two prescriptions and applying a proton RBI of 1.1 correspond to *D* = 110 Gy (RBE) and *D* = 44 Gy (RBE), respectively. Proton beam dosimetry was performed in a solid water RW3 phantom (PTW) with a Markus-type chamber (PTW) connected to the Unidos Webline electrometer (PTW). The ionization chamber was positioned at the isocenter of the plan.

#### 2.1.3. Dose Prescription for IMPT Brain Irradiations

Brain tumors and CNS tumors are, besides leukemia and lymphoma, the most common cancers in children, and proton radiotherapy is an important radiation modality for treating them. In clinical practice, proton therapy is performed in multiple fractions depending on the tumor location and patient age with doses in the range of 40–65 Gy and 1–2 Gy/fraction (3, 4, 6). Different field arrangements are used with lateral fields, vertex fields or a multi-field combination of these orientations. PT is also a recognized method of cerebral arteriovenous malformation (AVM) treatment (usually treated with GK and LINAC stereotactic irradiation) with the advantage of minimal dose delivered behind the distal edge of the proton beam (35). Medium-size AVMs (diameter 3–6 cm) and large AVMs (diameter > 6 cm) are typically treated with a total dose of 21–25 Gy (RBE) (36), 12–28 Gy (RBE) (37), or 36–46.2 Gy (RBE) (38).

### 2.2. Comparison of Proton and Photon Radiotherapy

Out-of-field doses following (IMPT) were compared with previous measurements carried out within EURADOS WG9 for different photon radiotherapy techniques: IMRT, 3D-CRT, and GK treatment. Photon measurements were performed on a Varian Clinac 2300 linear accelerator (LINAC) in the Centre of Oncology, Krakow, Poland, and on a Leksell Gamma Knife (GK) (Model 4 C, Elekta Instruments, Stockholm, Sweden) at University Hospital Centre Zagreb, Croatia (33, 34). It is important to note that phantoms and detectors which were used in previous experiments as well as target location were the same as for proton irradiations. Treatment plans for both photon (3DCRT, IMRT, and GK) and proton modalities (IMPT) simulated a realistic clinical situation and the typical planning protocols used in the participating radiotherapy centers. The irradiation conditions for different radiotherapy techniques are shown in **Table 2**.

**Table 2:** Details of irradiation set-up for different RT techniques investigated by Eurados WG9.

Technique	Machine/Site	Irradiation plan	
IMPT	IBA Proton Therapy System - Proteus 235, Krakow, Poland	2 coplanar beams (140˚ and 270˚) *D*_T_ = 100 Gy (luminescent detectors) *D*_T_ = 40 Gy (track detectors)	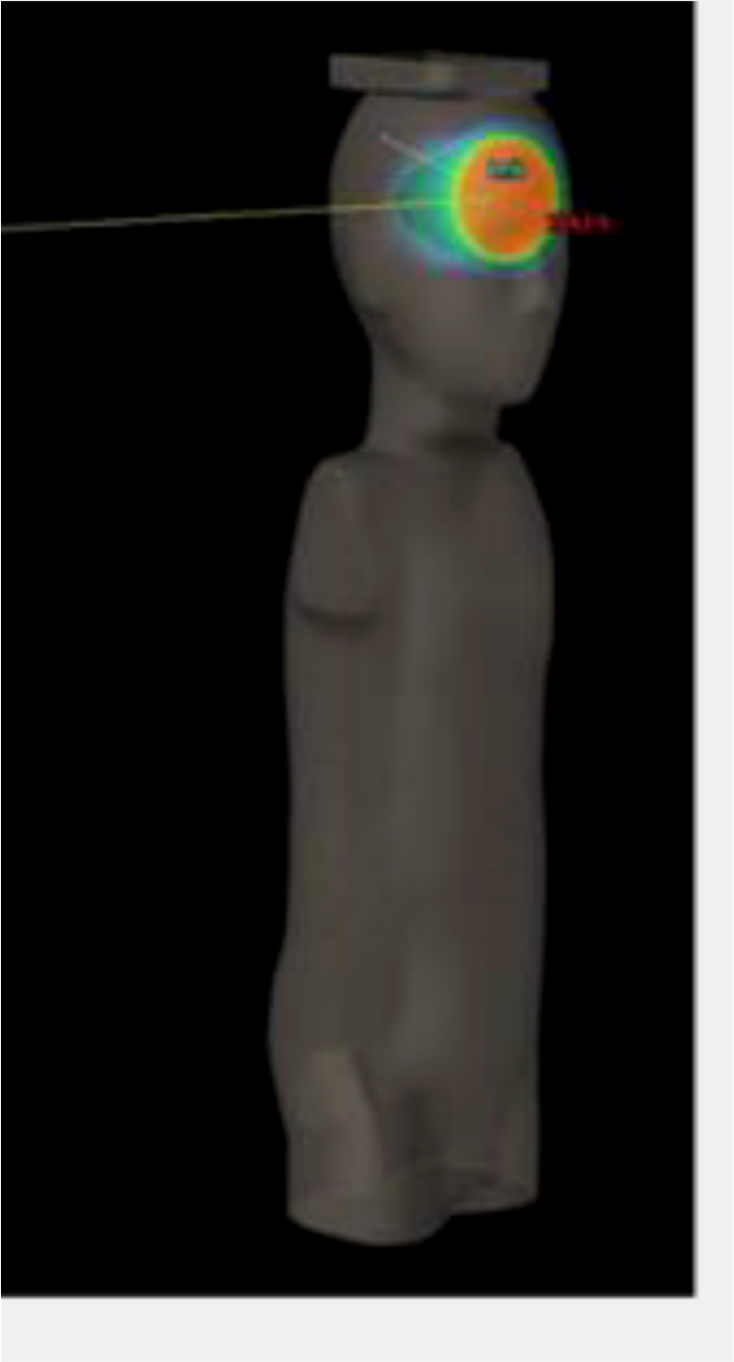
3D-CRT(33)	Varian Clinac 2300,Centre of Oncology Krakow, Poland	3 non-coplanar beams (6MV) 336 MU Dynamic and mechanical wedge *D*_T_ =2 Gy	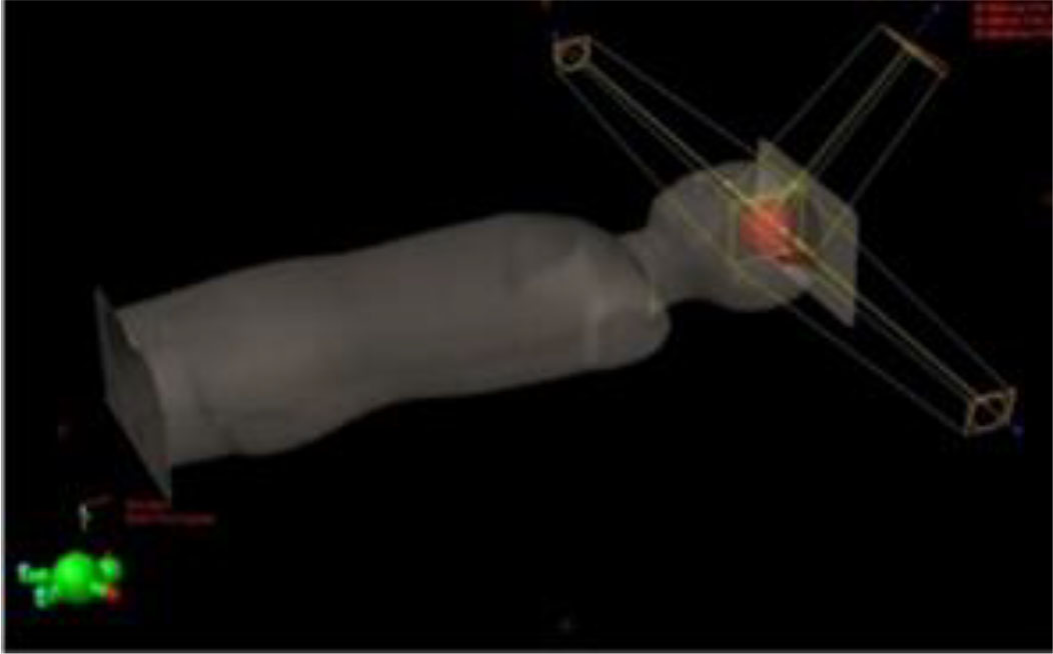
IMRT(33)	Varian Clinac 2300, Centre of Oncology Krakow, Poland	9 coplanar beams (6MV) 443 MU *D*_T_ =2 Gy	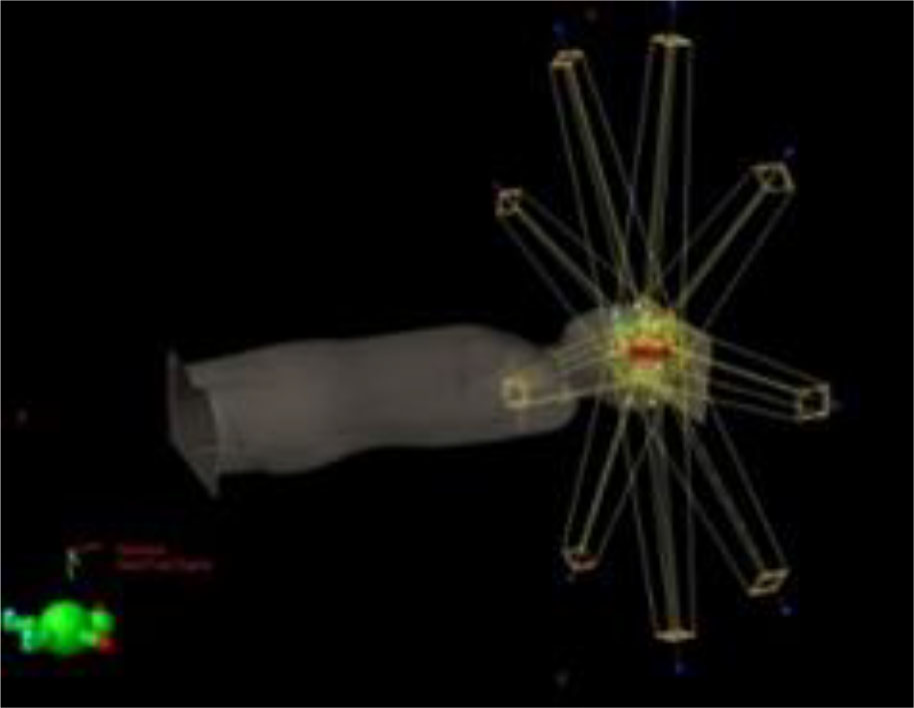
GammaKnife (34)	Leksell GK (model 4C), University Hospital Zagreb, Croatia	Collimated beams from array of Co-60 sources; 18 mm collimator *D*_T_ =4.1 Gy	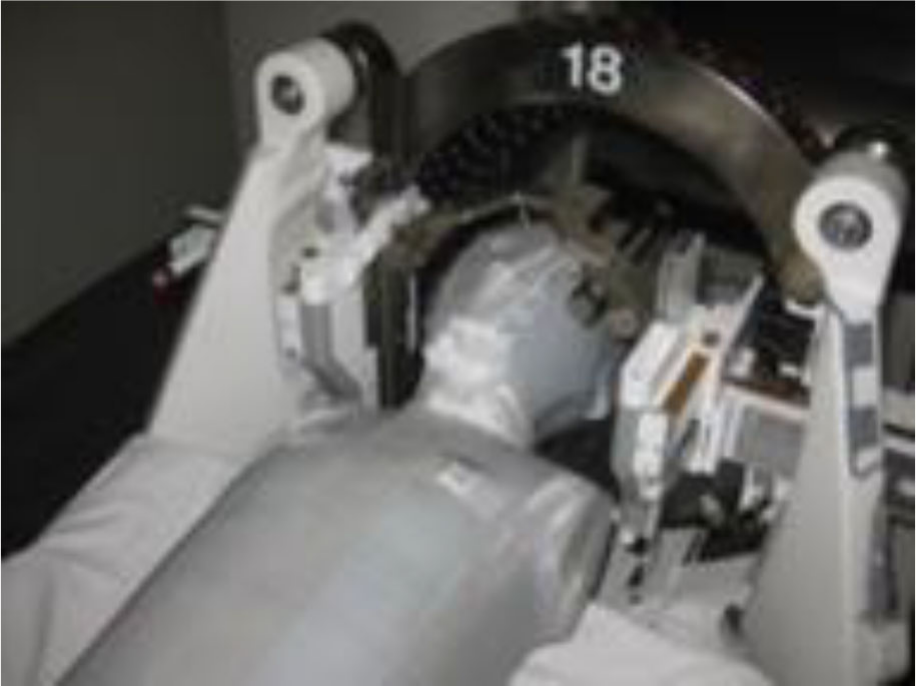

### 2.3 Dosimetry Systems and Dose Calculations for Out-of-Field Dose Estimation

In conventional radiotherapy with high-energy X-rays, out-of-field doses are usually expressed in terms of neutron dose equivalent or equivalent dose in organ and gamma-ray-absorbed dose. In proton therapy, an additional contribution to out-of-field dose comes from scattered protons and from charged particles produced from nuclear reactions. Luminescence detectors, as thermoluminescent (TLDs) or radiophotoluminescent detectors (RPLs), are used for measurements of the gamma radiation component; however, they are sensitive to all types of ionizing particles. Their sensitivity differs depending on the detector type and isotopic composition. For example, for LiF-based TLDs the sensitivity to neutrons depends on the relative concentrations of Li-6 and Li-7 and on the neutron energy. Li-6-enriched TLDs are very sensitive to thermal neutrons due to their high 6Li(n,a)3H cross section for thermal neutron while Li-7-enriched TLDs, such as MTS-7, have a very low sensitivity to neutrons. The response of RPL dosimeters to neutrons is even lower than for TLDs enriched with Li-7.

Track detectors are used for measurements of the neutron component of out-of-field doses. The advantage of PADC detectors is their practical insensitivity to photons, but they may register not only secondaries from nuclear reactions with neutrons but also slowed-down protons. The neutron dose is expressed in terms of dose equivalent, to reflect their biological effect and allow a comparison among results. For the current application of dosimetry in proton therapy, the equivalent dose in organ can be assessed by the average of dose equivalent in representative points of the organ. As shown in the publication by Romero-Expósito et al., the use of quality factor Q or radiation weighting factor *w_R_* in the calculation of the dose equivalent in a point led to similar results and neutron doses evaluated using both factors are comparable with differences below 12% (39).

The response of different types of detectors used in this study was characterized for mixed radiation fields induced by proton pencil beams in previous papers published by EURADOS WG9 (22, 29). Here we summarize the most relevant aspects of the dosimetry systems used.

#### 2.3.1 Luminescence Detectors Used for Non-Neutron Out-of-Field Dose Measurements

The basic principles of the RPL and TL dosimetry methods and their characteristics, applicability, and calibration procedures were described in the paper previously published by EURADOS WG9 (40). In the current study, we used data from TL MTS-7 detectors (manufactured by IFJ PAN, Poland) and RPL detectors (GD-352M, manufactured by AGC Techno Glass (41).

TL and RPL dosimeters were calibrated with a 60-Co source in terms of kerma “free in air”, *K*air (*K*air was then converted to absorbed dose to water, *D*w), or directly in terms of *D*w as described in Knežević et al. (40). Relative standard uncertainties (1 SD in %) of the determined dose for RPLs (GD-352M) and TLDs (MTS-7) were 2.1% (for 1 mGy–2 Gy) and 2.7% (below 1 mGy) and 2.9% (for 2 mGy–5 Gy) and 4.2% (below 12 mGy), respectively (28, 39).

The out-of-field doses in proton therapy in the proximity of the target are dominated by secondary protons. Further from the target, the contribution from protons decreases, and the contribution from secondary neutrons and photons produced through inelastic and non-elastic nuclear interactions becomes dominant (42). Results presented in this paper are measured outside the primary proton radiation field (minimal distance from the field edge is approximately 5 cm), in the mixed field of secondary protons, neutrons, and gamma radiation. For measurements in a mixed radiation field, the sensitivity to different radiation components is an important issue and should be considered. MTS-7 detectors (LiF : Mg, Ti) contain almost pure (99.9%) ^7^Li and have a greatly reduced response to thermal neutrons (43). RPL dosimeters of type GD-352M contain a filter for compensation of energy dependence and have negligible response to neutrons (44). In this study, RPL dosimeters were chosen for organ dose measurements based on their lower sensitivity to neutrons in comparison to MTS-7. It should be noted that both TLDs and RPLs also measure the contribution from scattered and secondary protons (22, 29, 45). Moreover, it is not possible to distinguish the signal from protons from the signal from photons. For this reason, for doses measured with RPL detectors we use the term “non-neutron dose” to express the fact that RPL detectors register not only gamma rays but also to a limited extent neutrons and some charged particles. Measured non-neutron doses were normalized to the physical target dose *D*_T_.

#### 2.3.2 Track-Etched Detectors Used for Out-of-Field Neutron Dosimetry

Measurements of secondary neutrons were performed with two types of poly-allyl-diglycol carbonate (PADC) track-etched detectors, which relied on a different method for the calculation of the neutron dose equivalent, *H*_n_ (mSv). Type I track detectors (type HARZLAS TD-1, Nagase Landauer Ltd., Japan) use the relationship between the parameters of etched tracks and LET (46). Type II track detectors (Intercast Europe S.R.L., Parma, Italy) include a set of converters (polyethylene, Makrofol, and nylon) specifically designed to make the detector sensitive to neutrons from thermal to high energy range (47, 48). A weighted average of the fluence response factor can be evaluated from the specific response factor and the fraction of neutrons arriving to the point in each energy range (thermal, epithermal, evaporation, and high energy) (39). The assessment of neutron dose equivalent is then performed from neutron fluence following the procedure described in Romero-Expósito et al. (2016) (39). The overall uncertainties for both detector types are at the level of ~ 20%. More details about the detectors and their calibration can be found in the previously published papers (26, 46, 49). Type II track detectors were used only in the 5-year-old phantom with specially designed PMMA slices, which allowed the insertion of detectors inside the phantom at positions corresponding to 11 organs (thyroid, lungs, sternum, heart, liver, kidneys, stomach, intestines, bladder, ovaries, and testes) covering distances from the tumor in the range from approximately 6 to 40 cm from the isocenter.

The neutron contribution determined with PADC detectors is expressed as neutron dose equivalent, and the results are normalized per target dose (mSv or µSv/Gy). The measurements with track detectors were limited to selected positions and distances (up to approximately 40 cm from the isocenter). In order to compare with the non-neutron component and with out-of-field doses for other radiotherapy modalities, results were extrapolated basing on the curve fitted to the experimental data. Neutron dose equivalents presented in this paper for distances from approximately 30 to 65 cm were calculated from the abovementioned fit.

### 2.4 Calculation of Total Out-of-Field Dose

Out-of-field doses following intensity-modulated proton therapy (IMPT) obtained in this study were compared with doses for different photon radiotherapy techniques (IMRT, 3D-CRT, GK) previously measured by EURADOSWG9 (33, 34). For comparison purposes for IMRT, 3D-CRT, and GK, the photon dose equivalent was calculated by multiplying measured photon dose, *D* (mGy), by the quality factor Q = 1. In the part of the paper where a comparison of measured doses for all irradiation techniques is shown, a total dose equivalent term was used for IMPT results. Total dose equivalent is the sum of the neutron component extrapolated from track detector measurements and the non-neutron component measured with RPL detectors.

## 3. Results

### 3.1 Out-of-Field Dose in Proton Spot Scanning Radiotherapy

#### 3.1.1. Out-of-Field Doses as a Function of Distance From the Isocenter

In this section, results are presented as function of distance from the isocenter. For each dosimeter, the distance was calculated from the middle of the detector to the center of the spherical tumor. Results are normalized to the target dose deposited at the isocenter, i.e., the center of the target volume.

**Figure 2:**
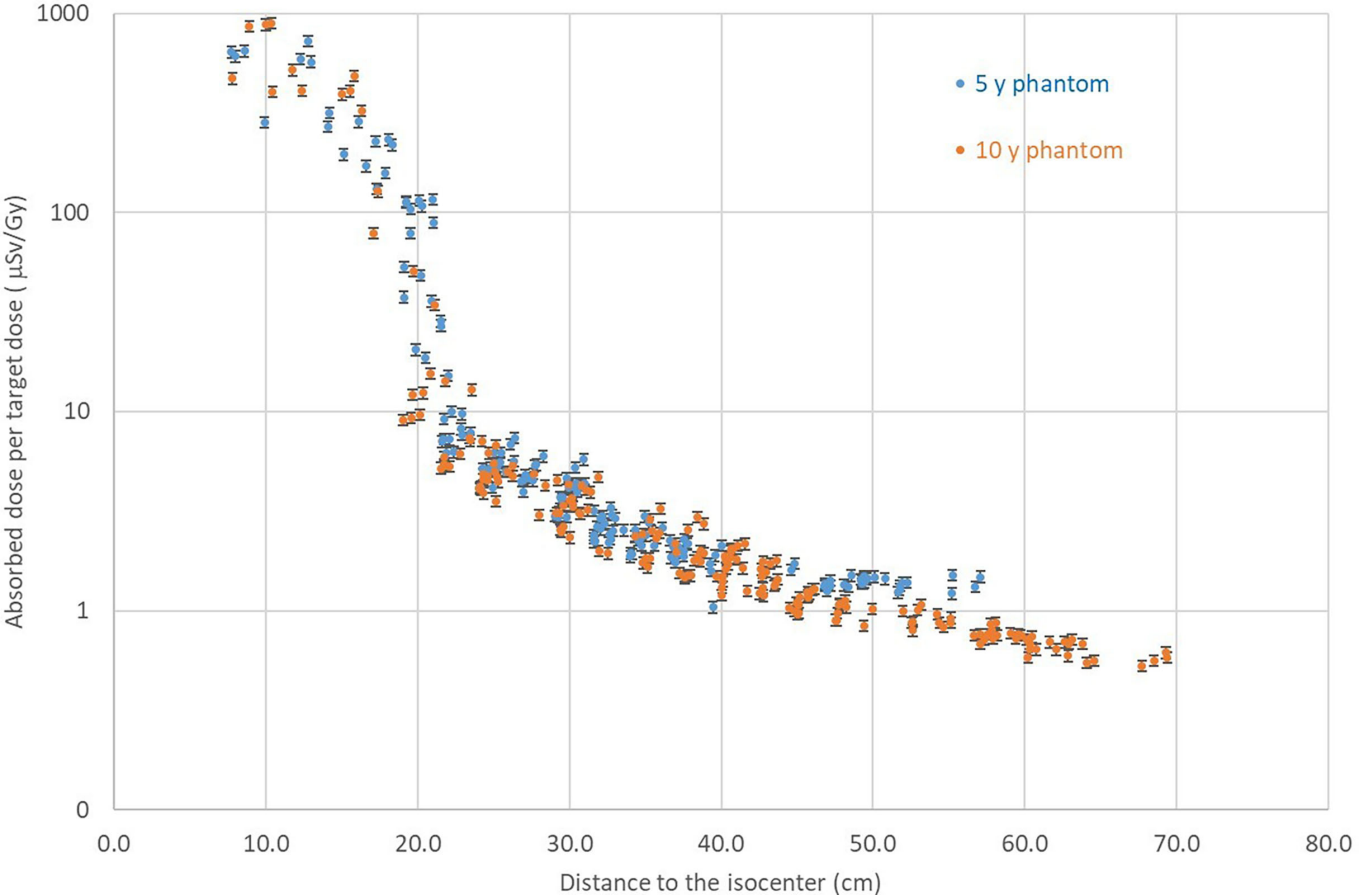
Comparison of non-neutron doses measured with RPL detectors for the 5- and 10-year-old phantom as a function of the distance from the isocenter.

**Figure 2** shows the non-neutron doses (obtained with RPL detectors) as a function of distance for 5- and 10-year-old phantoms. The results indicate that non-neutron doses as a function of the distance from the isocenter are comparable for 5- and 10-year-old phantoms and that the size of the phantom does not have a significant influence on the attenuation of non-neutron radiation. For both phantoms, non-neutron doses increase significantly in the proximity of the target, due to the presence of secondary and scattered protons, which can reach up to 15 to 20 cm from the isocenter and contribute to the detector signal (**Figure 2**).

**Figure 3:**
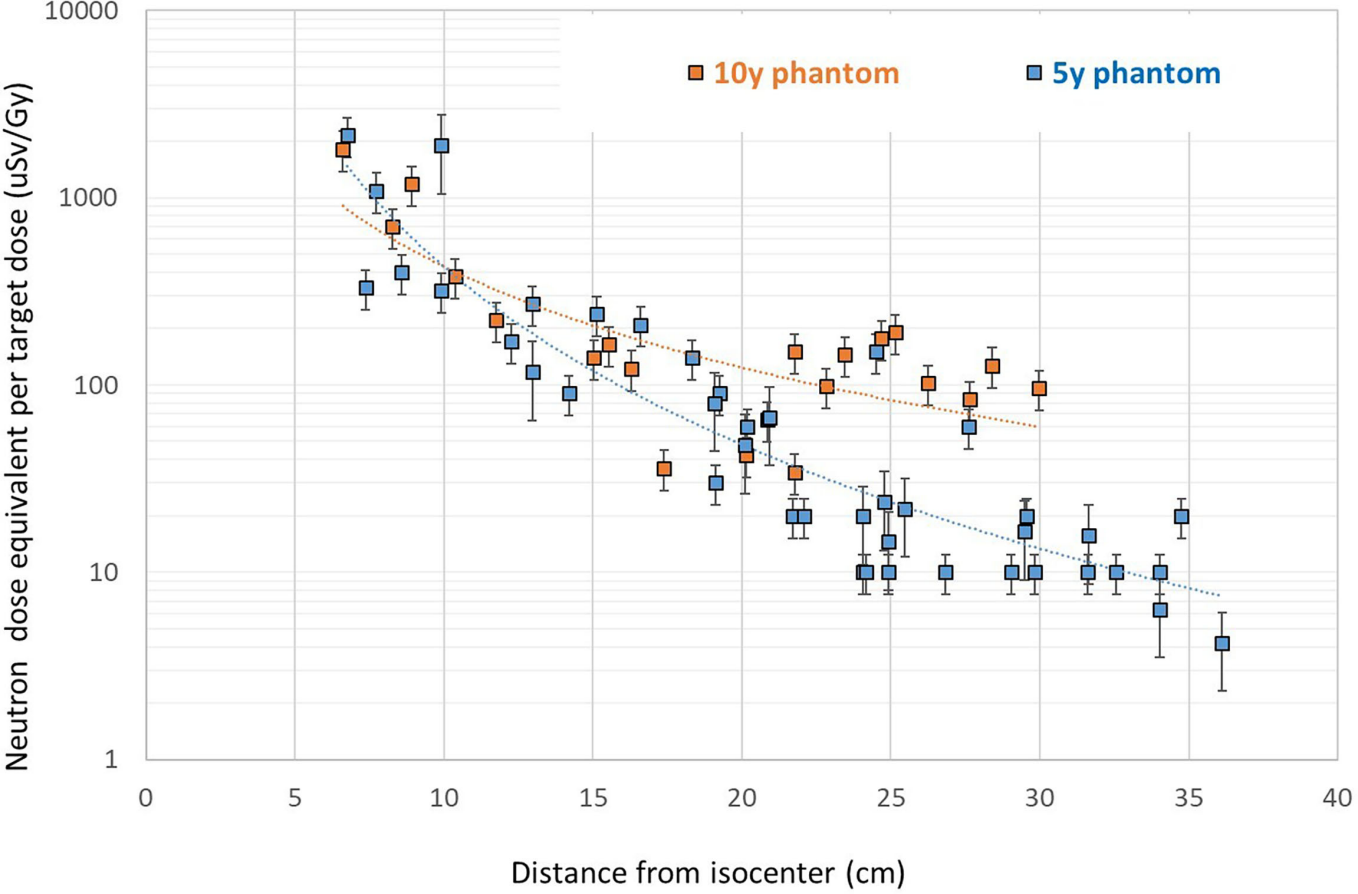
Neutron dose equivalent for the 5- and 10-year-old phantoms measured with PADC detectors. Error bar represents overall uncertainty of the track detectors.

Results for the neutron component measured with track detectors as function of distance are presented in **Figure 3**. A comparison of neutron doses for the 5- and 10-year-old phantom shows slightly higher neutron doses measured in the 10-year-old phantom. The comparison was performed for a selected number of positions due to the large dimensions of type II PADC detectors and the need for dedicated holders.

**Figure 4:**
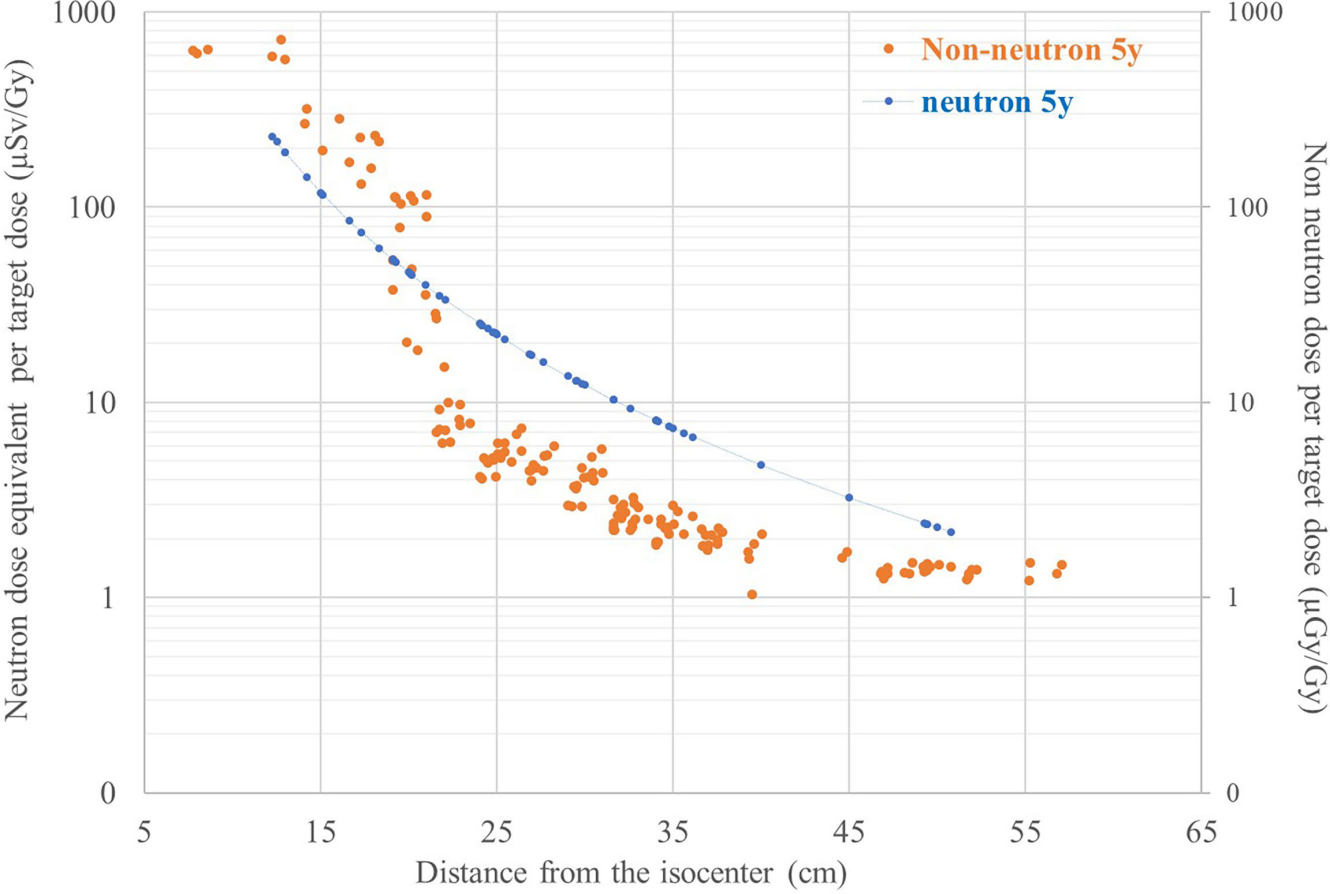
Comparison of neutron and non-neutron dose per target dose as function of the distance from the isocenter for the 5-year-old phantom.

The comparison of neutron doses and non-neutron doses for the 5-year-old phantom is shown in **Figure 4**. The neutron dose equivalent for the 5-year-old phantom was measured with two types of track detectors placed in different positions in the phantom. The agreement between them, taking into account the difference in calibration, calculation methodology, size, and location, is acceptable. As explained in Section 2.3.2 data, **Figure 4** presents extrapolated values of neutron dose equivalent based on the curve fitted to the measurements with two types of track detectors. As shown in **Figure 4**, close to the target, neutron doses are lower than secondary non-neutron doses. This again may be explained by the increase in signal measured by RPL detectors due to a contribution from secondary and scattered protons. It is expected that this contribution decreases strongly with distance. It was shown that at about 150 mm from the isocenter, protons contribute much less to the signal than gamma radiation (22). Further away from the target, outside the range of scattered protons the secondary neutron dose becomes larger than doses measured with RPL detectors. Both neutron and non-neutron doses decrease with distance from the target as, for PBS, secondary radiation is produced mainly by the interaction of protons with the patient body and lesser extent with the beam delivery system.

#### 3.1.2. Out-of-Field Non-Neutron and Neutron Organ Doses

**Figure 5:**
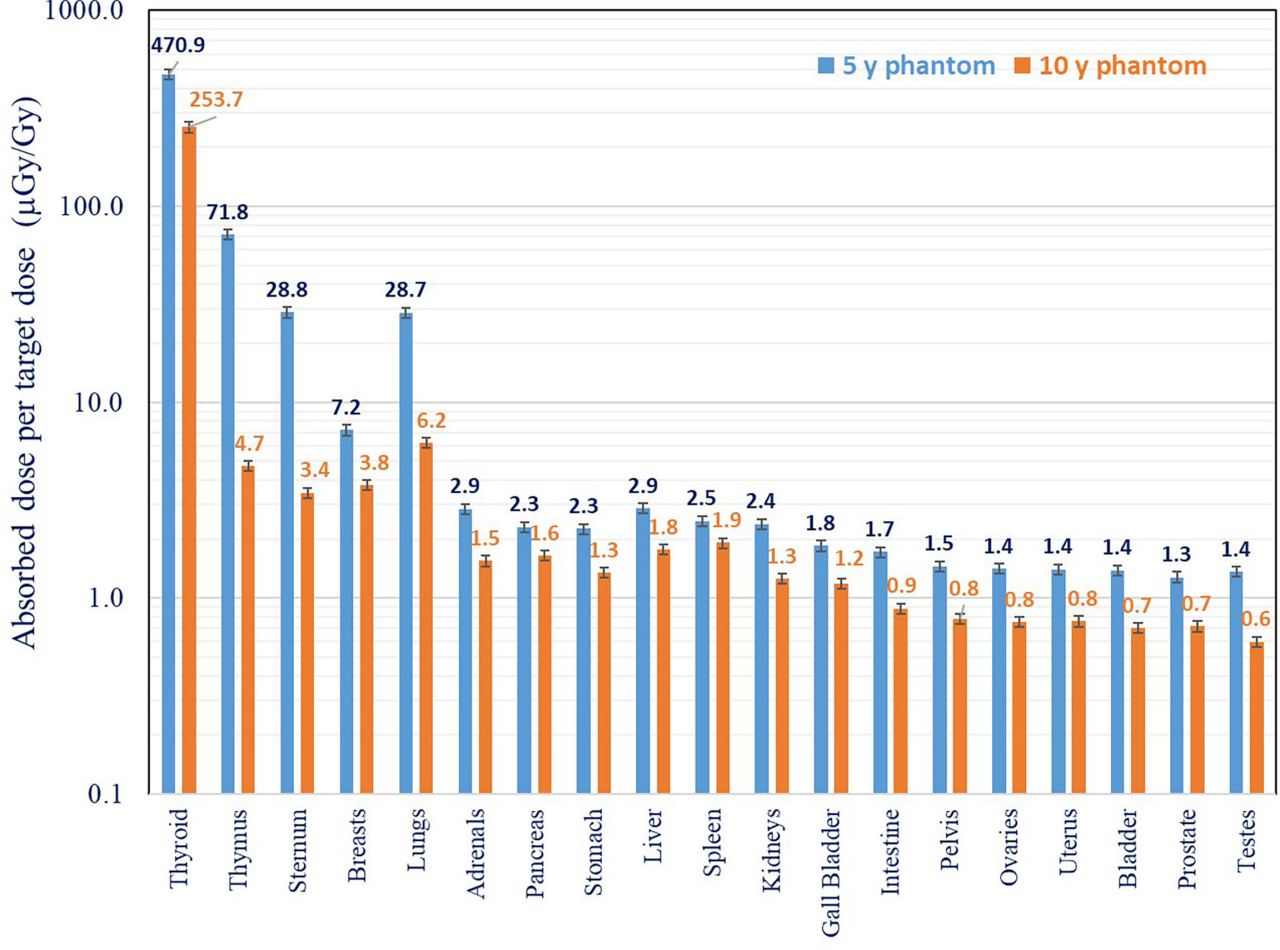
Comparison of non-neutron out-of-field organ doses for 5- and 10-year-old phantom. Measurements were performed with RPL detectors.

**Figure 6:**
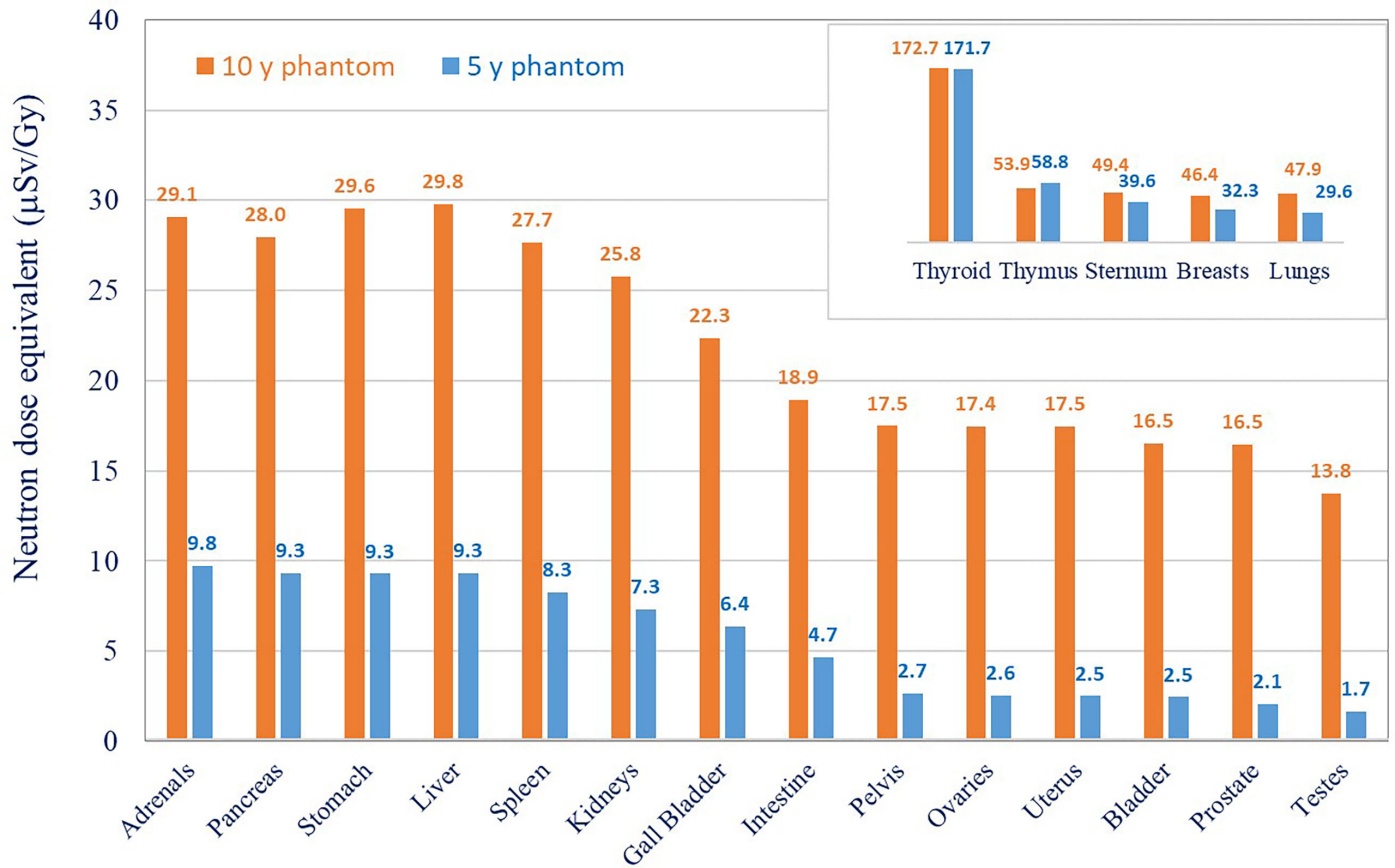
Neutron dose equivalent in different organs for 5- and 10-year-old phantom. Results were extrapolated from data the measured with track detectors.

In **Figures 5** and **6**, the neutron and non-neutron organ doses, obtained with track and RPL detectors, respectively, are shown for 5- and 10-year-old phantoms. The results are normalized to the target dose deposited at the isocenter. Organ doses are calculated as average values of all detectors placed in the specific organ. **Figure 5** shows that non-neutron organ doses are on average three times higher in the 5-year-old phantom when compared to the 10-year-old phantom. The reason is that distances between organs and target in the 5- and 10-year-old phantom are different. In the smaller (5-year) phantom, organs are closer to the target and consequently to the main source of secondary radiation. For both phantoms, as non-neutron doses are increasing rapidly in the proximity of the target, doses for organs located close to the target are higher when compared with organs located distantly. The secondary non-neutron doses for the 5-year phantom ranged from about 0.47 mGy/Gy closer to the field edge (13 cm from the isocenter) to 1.5 µGy/Gy (50 cm from the isocenter) for thyroid and testes, respectively. For the 10-year-old phantom, secondary non-neutron doses ranged from 0.25 mGy/Gy (15 cm from the isocenter) to 0.6 µGy/Gy (70 cm from the isocenter) for the thyroid and testes, respectively. For the full treatment course delivering 54 Gy (RBE) to the target volume, this would correspond to approximately 14 mGy and 32 mGy for thyroid and testes, respectively.

Organ neutron dose equivalents measured and fitted for different organs are shown in **Figure 6**. Organ neutron dose equivalents measured in the 10-year-old phantom are higher in comparison to the 5-year-old phantom. The difference increases with the distance from the isocenter varying by a factor of 1.5 in breasts, 3 in the liver to the largest difference observed for bladder, ovaries, and testes (an average factor of 7). The neutron dose equivalent in the 5-year-old phantom was 172 µSv/Gy, 59 µSv/Gy, 39.6 µSv/Gy, and 2.5 µSv/Gy for the thyroid, thymus, sternum, and bladder, respectively, and that in the 10-year-old phantom for the same organs was 173 µSv/Gy, 54 µSv/Gy, 50 µSv/Gy, and 17 µSv/Gy. *H*_n_ ranged from 1 mSv/Gy close to the field edge to the 0.01-mSv/Gy 30-cm distance from the isocenter. There are several possible explanations for the difference in neutron organ doses in 5- and 10-year-old phantoms. Bone density varies significantly with age, especially for children. Pediatric models of CIRS phantoms use bone equivalent materials, which mimic bone tissue composition and density related to age. Neutron interactions with tissues of higher density can enhance detector signals in the 10-year-old phantom. Also, the dimensions of the phantom are different, and the size influences both proton beam energy and neutron interactions. For the 10-year-old phantom, a slightly higher contribution of more energetic protons was needed to cover the target, and then more neutrons are produced. On the other side, high-energy neutrons have less probability of interaction in a smaller volume, and if they are not slowed down enough, they can escape from the phantom without interaction. Conversely, in a larger phantom, the probability of interactions is higher and then the dose can be higher.

#### 3.1.3. Out-of-Field Total Organ Dose

**Figure 7:**
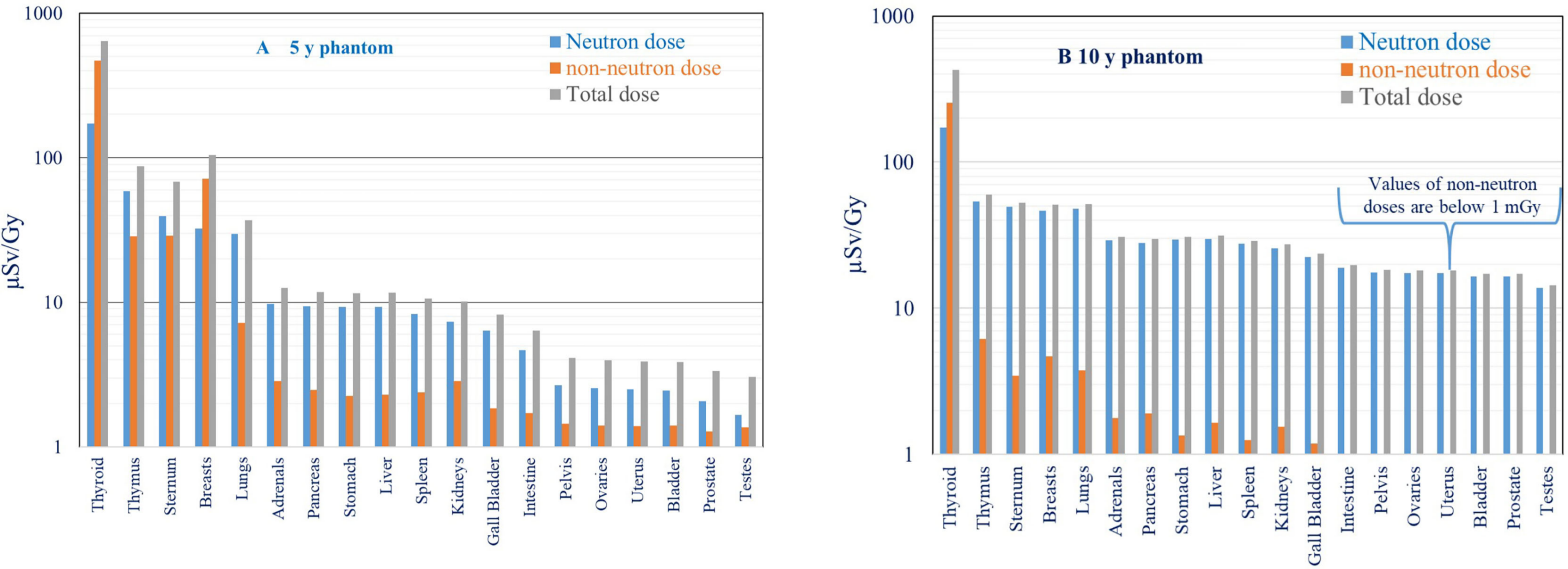
**(A, B)** Comparison of neutron, non-neutron, and total equivalent organ doses (µSv/Gy) for 5- **(A)** and 10-year-old **(B)** phantoms.

The comparison of neutron doses, non-neutron doses, and total organ doses (expressed as the sum of neutron and non-neutron doses) is shown in **Figures 7A, B** for both phantoms. As explained in Section 2.3.2, the neutron organ doses are calculated from fitting measurement results obtained with two types of track detectors. Due to the contribution from scattered protons in the organs close to the target, neutron doses are lower in comparison to the secondary non-neutron doses. For organs further from the target, the neutron dose increases in comparison to the non-neutron dose and dominates in total dose (as explained in Section 3.1.1). Non-neutron doses measured in the thyroid at 13.5 and 16.6 cm from the isocenter are by factors of 3 and 1.5 higher in comparison to neutron doses for 5- and 10-year-old phantoms, respectively. For the organs further away from the field (intestine, bladder, ovaries, testes) in the 10-year-old phantom, the non-neutron doses are below 1 mGy and are not visible, as shown in **Figure 7B**. In the proton scanning beam, mean out-of-field total doses including neutron and non-neutron components range from 0.6 mSv/Gy (5-year), 0.4 mSv/Gy (10-year) in the thyroid to <0.01 mSv/Gy for both phantoms in the intestines, ovaries, bladder, and testes.

### 3.2 Comparison of Out-of-Field Doses for Different Radiation Therapy Modalities

**Figure 8:**
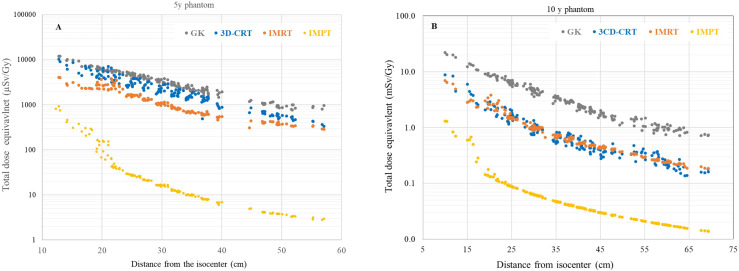
**(A, B)** Comparison of total dose equivalent organ doses for all irradiation techniques as function of distance from the isocenter for 5- **(A)** and 10-year-old **(B)** phantoms.

**Figure 9:**
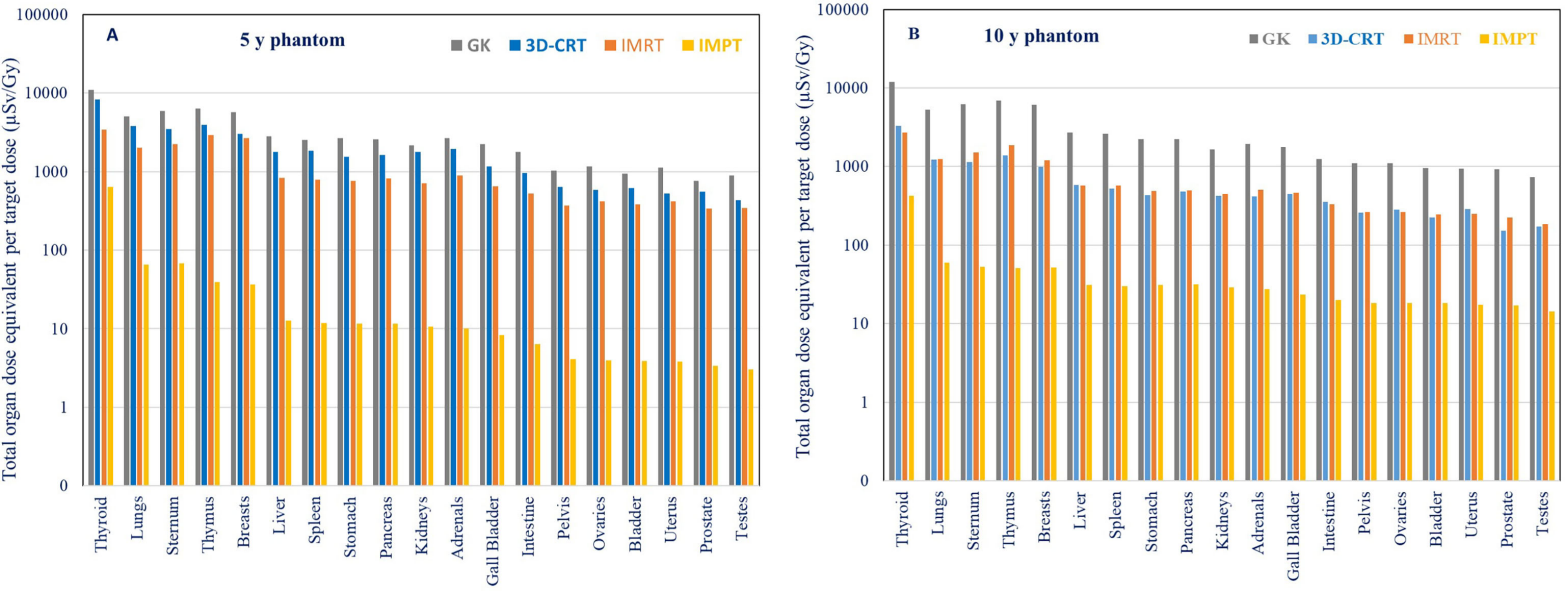
**(A, B)** Comparison of total doses in a 5-year-old phantom and a 10-year-old phantom for IMRT,3D-CRT, GK and IMPT. For IMPT, the total dose equivalent is taken as the sum of neutron and non-neutron contributions, and for the photon techniques, the total dose equivalent is simply the photon component.

Results obtained in this study were compared with out-of-field doses measured for different photon therapy modalities (**Figures 8** and **9**). In the previous experiments performed by EURADOS WG9, measurements of out-of-field doses in 5- and 10-year-old phantoms were performed for 3D CRT, IMRT, and GK radiotherapy (33, 43). In all experiments, the 5-cm-diameter brain tumor (PTV was 6 cm) was situated inside the left hemisphere of the head (intracranial tumor). Details of the irradiations for each technique are shown in **Table 2**. As shown in **Figures 8A, B**, a comparison of total dose equivalents measured as a function of distance from the isocenter is shown for 3D-CRT, GK, IMRT, and IMPT for 5- and 10-year-old phantoms. As the measurements with track detectors were performed only on limited positions, the results for distances from approximately 30 to 65 cm were calculated as described in Chapter 2.3.2.

A higher ratio of measured doses from 3D-CRT in comparison to measured doses with IMRT in the 5-year-old phantom compared to the 10-year-old phantom is shown in **Figures 8A, B**. This is explained in detail in a previously published paper by the use of a mechanical wedge for 5-year-old phantom 3DCRT treatment which increases out-of-field doses (33). Except for the eyes which were better spared during the GK treatment in comparison to 3DCRT and IMRT, out-of-field doses for more distant organs were higher up to factors of 2.8 and 4 times for GK compared to IMRT in 5- and 10-year-old phantoms, respectively. Results for IMPT show significantly lower out-of-field doses for both phantoms (**Figures 8A, B**) when compared to all three photon therapy techniques. The difference close to the target is at the level of one order of magnitude and more than two orders of magnitude further away from the target. The difference between three photon techniques and IMPT is more pronounced for 5-year-old phantoms. The ratio of different photon techniques in comparison to IMPT is as follows: 3DCRT/IMPT, GK/IMPT, and IMRT/IMPT are 120, 185, and 62 for the 5-year-old phantom and 14, 61, and 14 for the 10-year-old phantom, respectively.

The comparison of total out-of-field organ doses in 5- and 10-year-old phantoms for IMPT and 3D-CRT, GK, and IMRT is shown in **Figures 9A, B** and **Table 3**. For photon techniques (3D-CRT, GK, and IMRT), beam energies are below 10 MeV and the contribution of secondary neutrons can be neglected. Consequently, the total out-of-field dose is considered to be the out-of-field photon dose. For IMPT, the total dose equivalent includes both contributions from neutron doses based on measurements and calculations based on track detector data and non-neutron doses measured with RPL detectors.

**Table 3:** Comparison of measured total organ dose equivalent in selected organs for different techniques for the same brain tumor treatment in 5- and 10-year-old phantom.

5-year phantom						
Techniques	Total organ dose equivalent per target dose (mSv/Gy)					
	Thyroid	Thymus	Lungs	Liver	Bladder	Testes
**GK**	10.96	6.34	5.06	2.82	0.94	0.90
**3D-CRT**	8.28	3.93	3.80	1.78	0.62	0.43
**IMRT**	3.44	2.93	2.00	0.83	0.38	0.34
**IMPT**	0.64	0.04	0.06	0.01	0.004	0.003
**10-year phantom**						
**GK**	12.0	6.91	5.57	2.73	0.95	0.73
**3D-CRT**	3.26	1.38	1.22	0.58	0.22	0.17
**IMRT**	2.70	1.85	1.24	0.58	0.25	0.19
**IMPT**	0.42	0.05	0.06	0.03	0.02	0.01

In all cases, for both photon and proton radiotherapy, higher organ doses were measured for the 5-year-old phantom in comparison to the 10-year-old phantom, as expected due to the smaller distance from healthy organs to the irradiated target. As shown in the previous study, organ dose equivalents were on average 1.1, 1.6, and 3.0 times higher for the 5-year-old than for the 10-year-old phantom for GK, IMRT, and 3D CRT, respectively (32, 33). Non-neutron organ dose comparisons performed in this study for IMPT show on average 1.8 times higher doses for the 5-year-old phantom then for the 10-year-old phantom.

## 4. Discussion

Out-of-field organ dose measurements under realistic clinical conditions are important for validation and benchmarking of dose calculation methods and are also an important input for secondary cancer risk modeling. In the literature, there is little data on organ doses for child brain tumors irradiated with PBS. Moreover, for PBS, there are no studies with measurements of secondary radiation doses to specific organs under realistic clinical conditions inside pediatric anthropomorphic phantoms containing materials with realistic tissue densities.

The available data are mostly for passive scattering techniques which are associated with higher doses from secondary radiation in comparison to active technique due to the contributions to secondary radiation from beam formation elements (14, 27, 50, 51). In the paper by Gudowska et al., a review of secondary doses in ion therapy is shown but mostly for adult patients, or passive scattering. The paper showed large variations of secondary absorbed doses to healthy organs from 7 µGy to up to 2.4 Gy (per prescribed dose) (28). In the paper by Ardenfors et al., the out-of-field absorbed and equivalent doses in different organs calculated by MC simulations for a whole-body phantom (age 25) ranged from 60.36 µGy/Gy to 0.22 µGy/Gy and from 151 µSv/Gy to 0.63 µSv/Gy for thyroid and ovaries, respectively (25). These values are lower than doses measured in the current study due to a larger phantom size and consequently larger distances from the target in the published study. Moreover, Ardenfors et al. used one lateral proton field with energies between 60 and 97 MeV to cover the 133-cm^3^ target volume (25). In the current study, for both phantoms the PTV was 113 cm^3^ and two proton fields with energies between 70 and 140 MeV were used. Higher proton energies correspond to higher out-of-field doses (31). In the other study by Ardenfors et al., the organ absorbed doses calculated for a 6-year-old male patient for brain proton radiotherapy with a pencil beam scanning technique were 23 µGy/Gy and 0.8 µGy/Gy for the thyroid and bladder, respectively. The treatment plans were created with one lateral and one vertex field with energies 80–110 MeV and 92–124 MeV, and the planned target volume (PTV) was 24 cm^3^ (51). Out-of-field doses are increasing with proton energy, and primary field size (51 the difference in the PTV (24 cm^3^ vs 113 cm^3^) and maximum energy (124 MeV vs 140 MeV) may explain the differences between doses simulated by Ardenfors et al. and doses measured in the current study. Sayah et al. performed detailed simulations of secondary radiation doses for proton radiotherapy of pediatric patients treated for intracranial tumors using a passive scattering technique and reported averaged neutron equivalent doses for a 5-year-old patient of 1.79 mSv/Gy and 0.41 mSv/Gy for the thyroid and bladder, respectively (27). On the other hand, in the paper of Geng and al., neutron equivalent doses simulated for a proton pencil beam scanning technique for 14-year-old brain tumor patients were in the range of 100 µSv/Gy and 1 µSv/Gy for the thyroid and bladder, respectively (52). In turn, neutron equivalent doses simulated by Ardenfors et al. for a 6-year-old male patient for a brain proton PBS radiotherapy was 62 µSv/Gy and 2 µSv/Gy for the thyroid and bladder, respectively (51). Results obtained by both Geng et al. and Ardenfors et al. are similar to the data presented in this study, taking into consideration different field setups, size of PTV, and energies used in different studies. Organ dose equivalents for the 5-year-old phantom (with PTV 113 cm^3^) from this study ranged from 176 to 1.8 µSv/Gy and are in a good agreement with MC simulations performed by Ardenfors et al. (51), where neutron equivalent doses to organs ranged between 141 and 0.5 µSv/Gy for the 6-year-old male patient (with PTV 24 cm^3^).

When comparing different radiotherapy modalities presented in this paper, it can be seen that for IMPT with a typical treatment dose up to 2 (RBE) Gy in 27 fractions, the total absorbed doses (non-neutron + neutron component) are 32 mGy (5-year phantom) and 21 mGy (10-year phantom) in the thyroid, 1.77 mGy (5-year phantom) and 2.5 mGy (10-year phantom) in the breasts, and, on average for both phantoms, 0.4 mGy in the testes. This is significantly lower in comparison to the IMRT technique where for the full treatment the doses would be 169 mGy (5-year) and 133 mGy (10-year) for the thyroid, 131 mGy (5-year) and 59 mGy (10-year) for the breasts, and 17 mGy (5-year) and 9 mGy (10-year) for the testes. An additional comparison of total equivalent doses in selected organs for different techniques is shown in **Table 3**. The highest out-of-field organ doses (total dose equivalent) were measured for the GK technique (12 mSv/Gy in thyroid to 0.90 mSv/Gy in testes) while for IMPT the total dose equivalents were 0.42 mSv/Gy (10-year) and 0.65 mSv/Gy (5-year) in the thyroid and 0.003 mSv/Gy (5-year) and 0.01 mSv/Gy (10-year) in the testes. In the real clinical situation, the GK radiotherapy of a 5-cm-diameter target is performed for a large cerebral arteriovenous malformation AVM, where a dose of 30 Gy is delivered in five fractions (53). In this case, organ doses to the entire treatment are from 344 mGy in the thyroid, 177 mGy in the breasts, and 24 mGy in the testes on average for both phantoms. Based on literature findings, proton radiotherapy can be successfully used in treating intermediate- and large-sized AVMs (35). For the large-sized AVMs typically treated with protons, the prescribed dose is approximately 21–25 Gy ( (36). Consequently, total organ doses (averaged over both phantoms) based on measurements from this study would be 12, 1, and 0.20 mGy for the thyroid, breasts, and testes, respectively. However, it should be noted that in a standard clinical treatment, GK is used to treat much smaller target volumes than presented here.

There is no similar comparison of out-of-field doses between photon and proton radiotherapy available in the literature. Previous studies are mostly performed by MC simulations or using TPS calculations. Even if the same phantoms and similar PTV are used, there are always differences in the configuration of the radiation fields between different techniques and also between different facilities, leading to difficulties in explaining differences between out-of-field doses. Also, clinically used treatment planning systems are not aimed at an accurate calculation of out-of-field doses originating from secondary radiation (54, 55).

Measurements of out-of-field doses in realistic conditions presented in this study provide appropriate methodology and are important for second cancer risk calculations as well as input to analytical models for eventual clinical implementation.

Even though for the same brain tumor treatment, the tissues and organs received much lower total dose equivalents during IMPT in comparison to different photon techniques as shown in this study, it is nevertheless important to consider the second cancer risk estimations in order to make risk–benefit judgements. It is therefore essential to develop databases of assessed doses from secondary radiation to healthy organs outside the primary fields in order to accurately evaluate the long-term outcomes associated with proton therapy.

## 5. Conclusions

In this study, out-of-field organ doses were measured inside 5- and 10-year-old pediatric anthropomorphic phantoms for the treatment of a 5-cm-diameter brain tumor using intensity-modulated proton therapy (IMPT) and compared with previous measurements for three different photon radiotherapy techniques: IMRT, 3D CDRT, and GK. The results showed that non-neutron doses are higher in the 5-year-old phantom compared to the 10-year-old phantom due to increased proximity of organs to the target. Neutron doses are lower than non-neutron doses close to the target (factor of 4 in thyroid). At the same time, neutron doses become larger than non-neutron doses further away from the target (factor of 3–4). The total dose equivalent in proton therapy ranges from 0.6 mSv/Gy in the thyroid to <0.01 mSv/Gy in the gonad region, while for photon techniques the total organ dose equivalent ranges from 12 mSv/Gy in the thyroid to 0.22 mSv/Gy in the gonad region. Proton therapy results in lower out-of-field doses compared to 3D-CRT, GK, and IMRT techniques by one order of magnitude close to the brain and more than two orders of magnitude further away from the brain.

## Data Availability Statement

The raw data supporting the conclusions of this article will be made available by the authors, without undue reservation.

## Author Contributions

ŽK, LS, RH, and PO contributed to the conception and design of the study. The measurement data analysis and interpretation of neutron dose measurements were performed by IA, MD, MS-H, MÁC-P, CD, MR-E, IMR. The measurements with TLDs and RPLs, data analysis, and interpretation of the results were performed by ŽK, LS, MM, SM, NM, and MS-H. The design of the irradiation setup and preparation of radiotherapy treatment plans were performed by NM, LS, DK, KJ, and RK. ŽK wrote the first draft of the manuscript. LS and MS-H wrote sections of the manuscript. All authors contributed to manuscript revision, discussion, and approval the submitted version.

## Funding

KJ acknowledges the support of InterDokMed project No. POWR.03.02.00-00-I013/16. The work was partly supported by the POIR.04.04.00-00-15E5/18 project carried out within the TEAM-NET program of the Foundation for Polish Science cofinanced by the European Union under the European Regional Development Fund. IMR acknowledges the financial support from the Spanish Ministry of Science, Innovation and Universities (RYC2018-024043-I).

## Acknowledgments

This study was carried out within, and partly supported by, the European Radiation Dosimetry Group (EURADOS, WG9 Radiation Dosimetry in Radiotherapy).

## Conflict of Interest

The authors declare that the research was conducted in the absence of any commercial or financial relationships that could be construed as a potential conflict of interest.

## Publisher’s Note

All claims expressed in this article are solely those of the authors and do not necessarily represent those of their affiliated organizations, or those of the publisher, the editors and the reviewers. Any product that may be evaluated in this article, or claim that may be made by its manufacturer, is not guaranteed or endorsed by the publisher.
